# Application of High-Resolution DNA Melting for Genotyping in Lepidopteran Non-Model Species: *Ostrinia furnacalis* (Crambidae)

**DOI:** 10.1371/journal.pone.0029664

**Published:** 2012-01-11

**Authors:** FengBo Li, BaoLong Niu, YongPing Huang, ZhiQi Meng

**Affiliations:** 1 State Key Laboratory Breeding Base for Zhejiang Sustainable Plant Pest and Disease Control, Laboratory of Entomo-molecular Biology, Sericulture Research Institute, Zhejiang Academy of Agricultural Sciences, Hangzhou, China; 2 Key Laboratory of Insect Developmental and Evolutionary Biology, Institute of Plant Physiology and Ecology, Shanghai Institutes for Biological Sciences, Chinese Academy of Sciences, Shanghai, China; American Museum of Natural History, United States of America

## Abstract

Development of an ideal marker system facilitates a better understanding of the genetic diversity in lepidopteran non-model organisms, which have abundant species, but relatively limited genomic resources. Single nucleotide polymorphisms (SNPs) discovered within single-copy genes have proved to be desired markers, but SNP genotyping by current techniques remain laborious and expensive. High resolution melting (HRM) curve analysis represents a simple, rapid and inexpensive genotyping method that is primarily confined to clinical and diagnostic studies. In this study, we evaluated the potential of HRM analysis for SNP genotyping in the lepidopteran non-model species *Ostrinia furnacalis* (Crambidae). Small amplicon and unlabeled probe assays were developed for the SNPs, which were identified in 30 females of *O. furnacalis* from 3 different populations by our direct sequencing. Both assays were then applied to genotype 90 unknown female DNA by prior mixing with known wild-type DNA. The genotyping results were compared with those that were obtained using bi-directional sequencing analysis. Our results demonstrated the efficiency and reliability of the HRM assays. HRM has the potential to provide simple, cost-effective genotyping assays and facilitates genotyping studies in any non-model lepidopteran species of interest.

## Introduction

Insects in the order Lepidoptera (moths and butterflies), including the domesticated silkworm (*Bombyx mori*) and many destructive pests of agriculture and forestry, are among the most diverse and species-rich groups of insects [1]. They are primarily phytophagous caterpillars, and occur in most terrestrial habitats all over the world. Lepidopteran insects have significant impact on human society, but whose underlying mechanisms and consequences for genetic diversity remain to be fully explored. However, resolving genetic variation has been hampered by the relative scarcity of available genomic resources in the lepidopteran species, except the model species (silkworm) [2], [3]. In non-model organisms with limited genomic information, molecular markers have proved useful as a tool for revealing the nature and extent of genetic variation [4]. Thus, developing an ideal marker system will be invaluable for non-model lepidopteran organisms.

Many conventional marker systems have wide range of applications, but fail to be readily applicable to the lepidopterans. For example, microsatellites which are popular DNA markers have been widely used in many organisms including insects [5], [6], but for the lepidopteran species, the development of microsatellite markers has been extremely difficult. One reason is that the isolation of microsatellite loci is relatively laborious and expensive [7]–[11]. More importantly, the high genotyping error rate often hinders genetic analysis due to the presence of null alleles and multilocus microsatellites (microsatellite families) that have very similar flanking regions pervasive in lepidopteran genomes [10]–[14]. These difficulties have led lepidopteran geneticists to search for alternative molecular markers.

An alternative approach is to develop single nucleotide polymorphisms (SNPs). Owing to their advantages over microsatellites which includes lower error rates and higher abundance in the genome, SNPs have been widely used in human and model species and are becoming the markers of choice for applications in non-model organisms [15]–[17]. However, SNP discovery and genotyping by current methods remain yet expensive or labour intensive in non-model organisms [16]. Additionally, for lepidopteran non-model species, development of SNP markers conventionally by high-throughput genome or transcriptome sequencing can not circumvent the same difficulties as developing microsatellites, because the SNP loci obtained may reside in duplicated genes or repetitive regions, resulting in the null alleles and multilocus genetic markers. An attractive strategy is to develop SNP markers from genomic regions that encode single-copy genes. This single copy gene-based approach has been recently used in two lepidopteran species, the moth *Ostrinia nubilalis* [18] and the butterfly *Bicyclus anynana* [19]. Both studies show that SNP discovery targeting single-copy genes facilitates development of useful molecular markers in lepidopterans. The techniques for SNP genotyping, however, are not without some limitations. For *O. nubilalis*, the genotyping method by PCR-RFLP (Restriction Fragment Length Polymorphism) is limited to detect SNPs within restriction endonuclease sites, and technically laborious and expensive as it requires post-PCR processing, such as performing restriction digest reactions, preparing agarose gels, and running electropheresis. As for *B. anynana*, application of the Illumina Golden Gate platform to SNP genotyping requires multiple preparation steps and is relatively expensive due to the initial high cost of probe production [19], [20]. As our knowledge of lepidopteran genomes increases and more single-copy genes are identified, a more simple and cost-effective method for SNP genotyping will be highly desirable.

In clinical research and diagnostics, high-resolution DNA melting (HRM) analysis has proved to be a powerful post-PCR technology for SNP genotyping [21], [22], with huge potential of applications in other fields. HRM can distinguish single base variation based on the analysis of the melting curves and thus has the capability to genotype SNP loci without the burden of sequencing [23]–[26]. Genotype detection by HRM does not depend on the variant position within PCR products. All procedures of HRM analysis following PCR amplification take place in a closed-tube system within a few minutes, reducing risk of contamination and allowing a simple and rapid analysis [21], [23]. HRM has developed two common methods for genotyping. The most common is HRM analysis of small amplicons (SA-HRM), which involves the use of PCR primers that are designed to bracket the informative SNP and develop the amplicons of 40–150 bp in length. In SA-HRM, most single base changes within the small amplicons can be easily genotyped [24]. The second genotyping method is unlabeled probe HRM (UP-PCR), which needs unlabeled probes that are usually 20–35 bp in length and blocked at their 3′ end to prevent extension [27]. In UP-HRM, asymmetric PCR is used to produce a surplus of the strand complementary to a probe and then variations under the probe can be genotyped based on resulting melting profiles from a low-temperature region where the probe dissociates from the excess complementary strand. Owing to the obvious advantages, HRM analysis for SNP genotyping have been recently introduced to other fields ranging from plants [28]–[31] to fishes [32], [33], all demonstrating its ease of use, rapidity, and cost-effectiveness.

The aim of this study is to evaluate the effectiveness of HRM assays for SNP genotyping in the lepidopteran non-model species *Ostrinia furnacalis* (Guenée) (Lepidoptera: Crambidae). The *O. furnacalis* is among the most serious lepidopteran pests of maize (*Zea mays*) in Asian countries [34], [35] and has limited genomic information. The single-copy gene *Tpi* (triose-phosphate isomerase) previously described [36], was used for SNP discovery and HRM analysis, and HRM results were validated by bi-directional sequence analysis. The potential of the HRM assays for genotyping other non-model species of Lepidoptera was also discussed.

## Results

### SNP discovery by sequencing

To find SNPs for genotyping, DNA samples from 30 females (10 from each population) of *O. furnacalis* obtained from three different populations (Of_CU, Of_CC, and Of_NC) were amplified by previously described primers [36], [37] and then PCR products were directly sequenced in both directions to identify the point mutation or small insertion/deletion. Sequence analysis showed that no variation was found in the *Tpi* locus of all the sequenced samples from Of_CU and Of_CC populations, while two single-base mutations were identified in the Of_NC population. One SNP *rs84* was a G>A substitution located 84 bp downstream of the reference sequence of *tpi* (1463 bp in length) reported by [36], and ascertained in 2 of 10 samples from the Of_NC population. The other SNP *rs577* was a C>T substitution located 577 bp downstream of the reference and determined in three other samples. The samples identified by sequencing were considered as known female controls used for genotyping unknown samples in this study.

### Genotyping by SA-HRM

**Figure 1:**
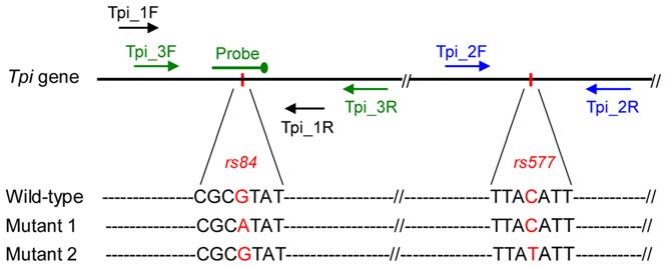
Details of the SNPs studied, including the primer and probe positions. The small vertical red bars indicate the newly identified SNPs in this study using the *Tpi* gene (Accession number DQ204987) as the reference. The primer and probe sequences are listed in Table 1.

To produce small amplicons, two primer pairs were designed to bracket the two polymorphic sites within the *Tpi* locus respectively summarized in Table 1 and Figure 1. Successful amplifications were achieved in all tested samples, including known female controls and 90 unknown female DNA samples (30 from each population). Rapid-cycle PCR of small fragments allowed PCR amplification in <30 min per run. Following PCR, SNP genotyping was directly performed using SA-HRM in a closed-tube system between 65°C and 85°C with a turnaround time of 1–2 min.

**Table 1:** Primer and probe sequences for HRM assays.

	SNP^1^	Primer/probe name^2^	Primer/probe sequence	Amplicon size (bp)	T_a_ (°C)^3^
SA-HRM					
	*rs84*: G>A	Tpi_1F	TGGATCCCAATGTTGAGGTAA	95	60
		Tpi_1R	ACACACTATCACTACCTATAGTCT		
	*rs577*: C>T	Tpi_2F	AAAGATGATCTTGTCGCCG	118	60
		Tpi_2R	GCATGGGCAACCTAAATATACAATAA		
UP-HRM					
	*rs84*: G>A	Tpi_3F	CCCAATGTTGAGGTAATGTTGATACATA	142	62
		Tpi_3R	CATGAGCCAGATAAATGGCGG		
		Probe	TCATTCGCATATTGAGATTGTG 4		

1 The SNPs were identified within *Tpi* gene by direct sequencing in this study. Details of the SNPs are displayed in Figure 1.

2 F, forward primer; R, reverse primer.

3 T_a_, optimized annealing temperature.

4 The underlined base was C3-blocked to prevent the 3′ end of the oligonucleotide from extending.

**Figure 2:**
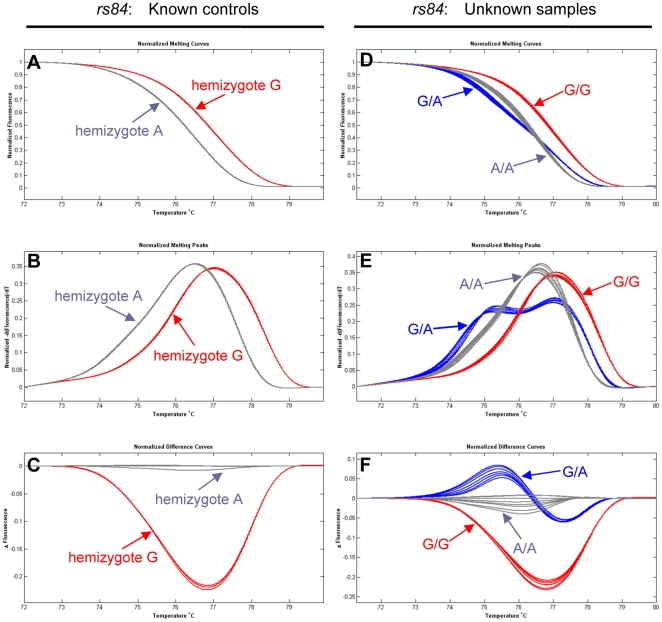
Genotyping of SNP *rs84* by SA-HRM. **A, D.** normalized melting curves. **B, E:** normalized derivative melting curves. **C, F:** normalized difference curves. Unknown samples were successfully genotyped by prior mixing with known controls. Arrows link genotypes with corresponding same color curves.

**Figure 3:**
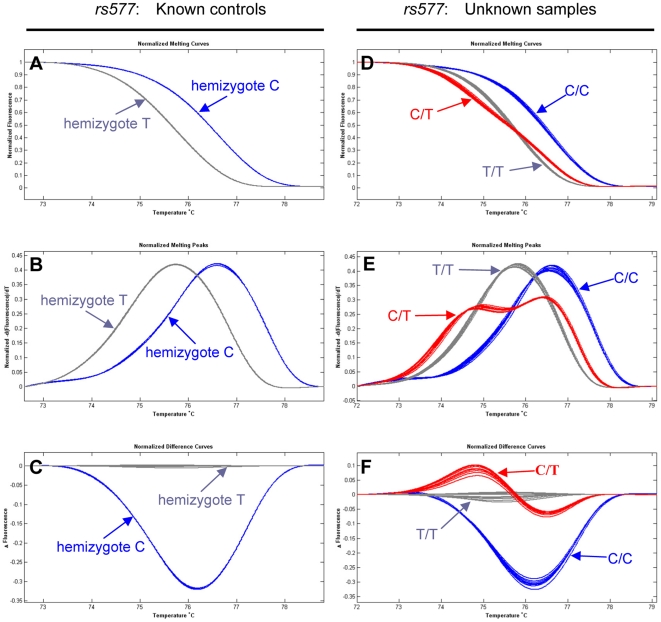
Genotyping of SNP *rs577* by SA-HRM. **A, D.** normalized melting curves. **B, E:** normalized derivative melting curves. **C, F:** normalized difference curves. Unknown samples were genotyped by prior mixing with known controls. Arrows link genotypes with corresponding same color curves.

For known female controls, the expected two hemizygous variants at each SNP site were resolved by SA-HRM as distinct normalized melting curves, melting peaks, and difference plots (Figure 2A, 2B, 2C and Figure 3A, 3B, 3C, respectively). The reproducibility of the SA-HRM assay was verified at multiple levels. The same DNA extracts were performed in duplicate or triplicate wells per run, and the small amplicons representing the same genotype were produced from DNA extracts of all 3 different populations of *O. furnacalis*. The HRM profiles for the same DNA preparations and the same sequences amplified from different populations were readily clustered together, allowing different genotypes to be consistently distinguished. The accuracy of genotype detection by SA-HRM was compared on the same samples identified using direct bi-directional sequencing in this study.

To further evaluate the effectiveness of SA-HRM for SNP genotyping, 90 unknown female DNA (hemizygous) samples were tested by mixing at the ratio of 1∶1 with the known wild-type female controls. If two hemizygous samples are not identical, mixing will generate a heterozygote that changes the shape of the melting curve [38]. Figure 2 and 3 displayed HRM curve analysis profiles of the SNPs *rs84* and *rs577*, respectively. In all cases, three different genotypes of a SNP could be readily distinguished by SA-HRM analysis. For the SNP *rs84*, 85 G and 5 A hemizygotes were identified in the 90 unknown females. For the SNP *rs577*, 82 C and 6 T hemizygotes were detected. All genotyping results were subsequently validated by bi-directional sequencing, demonstrating the reliability of the SA-HRM assay.

### Genotyping by UP-HRM

**Figure 4:**
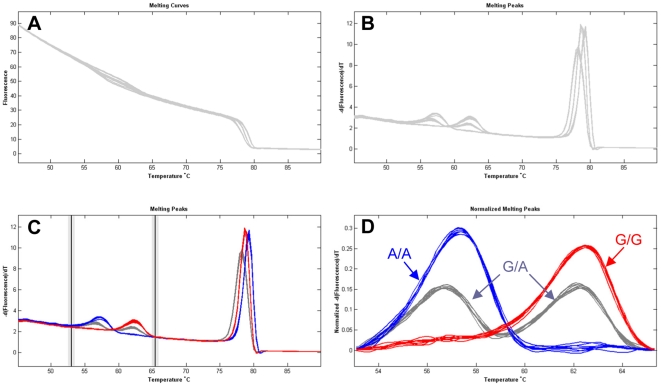
Genotyping of SNP *rs84* by UP-HRM. **A:** raw melting curve data. **B:** derivative melting curves. **C:** normalization of melting curve data. **D:** normalized derivative melting curves. Three groups are well distinguished: A/A in blue, G/G in red, and G/A in gray.

To assess the usefulness of UP-HRM for SNP genotyping, all the female DNA samples tested by SA-HRM were analysed using three oligonucleotides (a primer pair and one unlabeled probe) designed to target the SNP *rs84* (summarized in Table 1 and Figure 1). HRM curve analysis of the unlabeled probe is shown in Figure 4. All genotypes were distinct and easily resolvable. HRM profiles showed two melting transitions (Figure 4A, 4B, 4C), one for the unlabeled probe at a lower temperature and the other for the amplicon at a higher temperature. Homozygous samples were represented by a single probe melting peak, whereas heterozygous samples had two peaks corresponding to both hemizygotes separated by approximately 5°C (Figure 4D). The genotyping results were 100% concordant with that by the SA-HRM assay above and confirmed by bi-directional sequencing.

## Discussion

SNPs used widely in human and model organisms are becoming the marker of choice for applications in non-model species [16], [17]. For lepidopteran non-model organisms, SNP discovery targeting single-copy genes has proved to be an efficient approach [18], [19], but SNP genotyping by current techniques remains laborious and expensive. Here we first reported that HRM can provide a simple, sensitive and cost-effective technique for SNP genotyping in lepidopteran non-model species.

HRM analysis was very effective for SNP genotyping in the tested lepidopteran species *O. furnacalis*. For the demonstration set of 30 known and 90 unknown female samples, both SA-HRM and UP-HRM assays could readily resolve different genotypes of the SNPs identified in *O. furnacalis* (Figure 2, 3 and 4), because DNA amplicons that differ at a single nucleotide showed different HRM profiles. Although only the Z-linked gene *Tpi* was genotyped by HRM in this study, HRM can be applied for SNP genotyping regardless of whether target genes located on the Z chromosome or on an autosome in the lepidopteran species. Because hemizygous DNA of each unknown female sample was mixed 1∶1 (v/v) with that of a known wild-type female, each mixed sample has either homozygous or heterozygous DNA. Genotyping a mixed female sample by HRM assays is analogous to genotype a male sample or an autosomal gene. Compared to SA-HRM (Figure 3E), UP-HRM assay allowed a better discrimination of genotypes (Figure 4D), primarily due to the smaller target sequence having a more distinct difference in melting temperature (T_m_). This is valuable when there is a small change in T_m_ between different hemizygotes or homozygotes. Although only the two SNPs available were used for genotyping in this study, either SA-HRM [24] or UP-HRM [27] has the ability to genotype all SNPs when a known genotype is added. Sensitivity and specificity of both methods proved to be 100%, which were supported by our direct sequencing. The high reproducibility of HRM was also confirmed by our repeat experiments at multiple levels. These observations support the high value of HRM analysis for reliable genotyping in the lepidopteran non-model species *O. furnacalis*.

Although we have applied this technique to *O. furnacalis*, HRM analysis can be readily applied to other non-model species in the order Lepidoptera mainly due to its obvious advantages. The methods described in this study should work on DNA extracted from any other non-model organisms in this order. Since HRM is highly sensitive to detection of single nucleotide differences, all SNPs discovered in the non-model organisms can be genotyped by this technique. One major advantage of the technique is that there is no need for expensive and specific reagents other than a generic saturating DNA dye (e.g. LC Green plus) and the corresponding primer sets, so the reagent cost is very low [24], [27]. Another advantage is that there is no need for post-PCR processing, so the technique is very simple to perform on the HR-1 instrument. This instrument is a cost-effective solution especially when the sample size is relatively small. It can allow a fast turnaround time, as such, HRM analysis is rapid, being completed within 1–1.5 h per 40 samples. Since our optimization experiments are able to provide the best choice for PCR conditions, a conventional thermal cycler could also be useful for PCR amplification prior to HRM analysis. Our developed methodology on the HR-1 can also be readily applied without any modification, to high-throughput genotyping, by using commercially available multi-well (96 or 384 wells) instruments, such as the LightScanner 96 (Idaho Technology) and the LightCycler 480 (Roche). Therefore, as more single-copy genes are identified in more non-model species of Lepidoptera [18], [19], [39], the HRM technique for SNP genotyping will gain wide application due to the advantages mentioned above.

However, one limitation of the technique is the scarcity or unavailability of HRM instruments. While currently all real-time PCR instruments are designed to monitor fluorescence during DNA melting, not all of them are able to perform HRM assay [40]. Initial investment toward obtaining a suitable HRM instrument could be relatively high, but it is cost-effective in the long run due to the lower cost of HRM analysis than traditional methods [41]. While we utilized the HR-1 instrument for melting analysis, the HRM assays described in this study could also be carried out on other HRM platforms [40], thereby providing more choices of instruments for lepidopteran researchers.

In conclusion, we have demonstrated that HRM can readily and inexpensively resolve all the SNPs in *O. furnacalis*. In our tests of known controls and 90 unknown females of *O. furnacalis* from diverse geographical sources, the HRM results fully coincided with those obtained by our direct sequencing. Thus, HRM is a reliable, simple and cost-effective post-PCR technique that is suitable for genotyping *O. furnacalis* populations, and has the potential of wide application for genotyping studies (e.g. population studies, biodiversity analyses, linkage mapping, and species identification) in any other non-model species of Lepidoptera.

## Materials and Methods

### Insects

The culture of *O. furnacalis* was established from larvae collected in maize fields in central China, as described by Zhou *et al.* (2003) [42]. The cultured population of *O. furnacalis* (Of_CU) has been maintained at the Shanghai Institute of Plant Physiology and Ecology, Shanghai Institutes for Biological Sciences, Chinese Academy of Sciences. Two additional wild populations of *O. furnacalis*, Of_CC and Of_NC were sampled in April 2010 from maize fields in central China (CC) and northern China (NC), respectively. Egg masses of *O. furnacalis* were collected and transferred to State Key Laboratory Breeding Base for Zhejiang Sustainable Plant Pest and Disease Control, Laboratory of Entomo-molecular Biology, Sericulture Research Institute, Zhejiang Academy of Agricultural Sciences, China. The hatched larvae were reared on an artificial diet at 25°C under a 14∶10 light∶dark photoperiod and 70% relatively humidity in the laboratory. After pupation, moths from the cultured and two natural populations were preserved at −80°C until used for DNA extraction.

### DNA extraction

Genomic DNA of *O. furnacalis* was extracted from the adult virgin female moths using a DNeasy Tissue kit (Qiagen) according to manufacturer's instructions. DNA quality and concentration was assessed using a Beckman DU 640 spectrophotometer (Beckman Coulter, USA). DNA solutions were then prepared at a concentration of 10 ng/µl and stored at −20°C until use.

### SNP discovery

A DNA fragment of about 1600 bp including the entire coding region and introns of *Tpi* was amplified using a pair of primers ECBtpi_for1A (AGATGTCAAAATTCAACTCAG) and ECBtpi_rev5 (ATAGTTTACGAATTACGAGTT), as described by Dopman *et al.* (2005) [36] and Malausa *et al.* (2007) [37]. *O. furnacalis* has a female-heterogametic sex chromosome constitution (WZ in females, ZZ in males) [43]. Similar to its closely related species *O. nubilalis* [36], *Tpi* of *O. furnacalis* was also found to be located on the Z chromosome (unpublicated data). Thus, PCR products from only female moths were purified and directly sequenced using ABI PRISM3730 automatic DNA sequence systems (Sangon Biotech Co., Ltd., Shanghai, China) in order to give unambiguous sequence information. DNA sequences from 10 female moths from each population were edited using DNAstar Seqman software (DNASTAR Inc., Madison, WI, USA). One sequence (Accession number DQ204987) of *Tpi* in *O. furnacalis* reported by Dopman *et al.* (2005) [36], as a reference sequence, was involved in sequence alignment. Multiple sequence alignment with ClustalX 1.83 [44] was performed to identify potential SNPs. The newly determined unique sequences were deposited in GenBank (Accession numbers JF938131–JF938132)

### Primer and probe design

Primers and one probe were designed to have a T_m_ of between 59–65°C with LightScanner Primer Design software (Idaho Technology, Salt Lake City, UT). Pairs of primers were designed to flank the informative SNPs identified newly in this study. Amplicon size was kept below 150 bp, as differences among genotypes are easier to identify when the amplicons are shorter [45]. The designed primers were further checked for simulated fluorescent melting curves using a newly developed web-based tool uMelt (http://www.dna.utah.edu/umelt/umelt.html) [46]. The unlabeled probe was designed to sit over an SNP of interest and C3-blocked at its 3′ end to prevent extension in PCR reactions [47]. For each primer pair or combination of primer pair and one probe, the annealing temperature (T_a_) was optimized in a temperature gradient ranging from 58 to 72°C in order to amplify a specific PCR product. Gradient PCR was carried out using the EDC-810 gradient thermocycler (Eastwin, China) under the PCR cycling conditions described in the next section. The quality and quantity of PCR products were validated on a 1.5% agarose gel using standard methods. All primer and probe sequences are summarized in Table 1.

### PCR and HRM analysis

For the tested female DNA, both mixed and unmixed samples were prepared for *Tpi* (Z-linked) analysis, because hemizygous (or homozygous) variants are easier to be detected by mixing known and unknown DNA [38], [48]. Briefly, hemizygous DNA of unknown female samples was mixed 1∶1 (v/v) with that of known wild-type female DNA, which had been confirmed by direct sequencing during the SNP discovery. Typically, samples were prepared in duplicate, so one unknown female DNA (mixed and unmixed) and one known wild-type control were used for the following PCR and HRM assays.

PCR reactions were performed on the Rapid Cycler 2 instrument (Idaho Technology, Salt Lake City, UT) in a total volume of 10 µL containing 10 ng DNA template, 0.2 µM of each primer, 200 µM of each dNTP, 0.5 U of *Taq* polymerase (TransGen Biotech), 250 µg/mL bovine serum albumin (Sigma), and 1 µL LC Green plus (Idaho Technology, Salt Lake City, UT). Reaction mixtures were overlaid with 20 µL mineral oil to avoid evaporative losses ensuring melt curves uniformity. Thermal cycling conditions for SA-HRM were 95°C for 2 min, followed by 45 cycles of 94°C for 10 s, T_a_ for 10 s, and 72°C for 10 s. The final step was set as follows: one cycle of 95°C for 5 s, followed by rapid cooling to 25°C for 30 s, maximizing heteroduplex formation [45]. Asymmetrical PCR for UP-HRM was performed with the following changes to the PCR solution: 0.04 µM limiting primer, 0.2 µM excess primer, and 0.2 µM probe with a C3 blocker at the 3′ end (Sangon Biotech Co., Ltd., Shanghai, China). Asymmetrical PCR conditions were one cycle of 95°C for 2 min, followed by 45 cycles of 2 step temperature cycling of 94°C for 10 s, and T_a_ for 15 s, and the final step was set as above. All reactions were performed in duplicate for every sample, and all samples showing variant melting transitions were amplified for the third time.

After PCR, glass capillaries carrying samples were transferred to a high-resolution melting instrument (HR-1, Idaho Technology, Salt Lake City, UT) for HRM analysis. A temperature ramp rising at 0.3°C/s was used to acquire fluorescence data during DNA melting from 60°C to 90°C in SA-HRM and from 45°C to 90°C in UP-HRM. HRM curves were analyzed using the Lightscanner software (Idaho Technology, Salt Lake City, UT) following the protocols described previously [49]. The “Small Amplicon” module and “Unlabeled Probes” module in the “genotyping” mode of the software were utilized respectively for SA-HRM and UP-HRM analyses, both involving negative filter, normalization, and grouping. For genotyping by SA-HRM, difference plots of normalized melting curves were further obtained by taking the fluorescence difference of each curve from the average wild-type curve at all temperature points, with the ability to display slight differences in curve shape and T_m_ [23], [25]. Genotyping by UP-HRM was based on probe T_m_, obtained from negative derivative melting curve plots [27]. These analytical methods have been used previously for SNP genotyping [28], [32], [50], [51].

### Validation of HRM results

PCR products of all genotypes were purified and cloned into the vector pMD™18-T (TaKaRa). Positive clones were sent to Sangon Biotech Co., Ltd., Shanghai, China, for bi-directional sequencing. Sequences were analyzed as above by using the Lasergene software (DNASTAR Inc., Madison, WI, USA) and ClustalX 1.83 [44], in order to validate results of genotyping by HRM.
